# Extrachromosomal Circular DNA: Current Knowledge and Implications for CNS Aging and Neurodegeneration

**DOI:** 10.3390/ijms21072477

**Published:** 2020-04-02

**Authors:** Quratul Ain, Christian Schmeer, Diane Wengerodt, Otto W. Witte, Alexandra Kretz

**Affiliations:** 1 Hans-Berger Department of Neurology, Jena University Hospital, 07747 Jena, Thuringia, Germany; quratul.ain@med.uni-jena.de (Q.A.); christian.schmeer@med.uni-jena.de (C.S.); diane.wengerodt@med.uni-jena.de (D.W.); otto.witte@med.uni-jena.de (O.W.W.); 2 Jena Center for Healthy Ageing, Jena University Hospital, 07747 Jena, Thuringia, Germany

**Keywords:** alternative lengthening of telomeres (ALT), CNS aging, copy number variants (CNV), DNA repair, extrachromosomal circular DNA (ecc/ecDNA), genomic plasticity, neurodegeneration, telomere trimming

## Abstract

Still unresolved is the question of how a lifetime accumulation of somatic gene copy number alterations impact organ functionality and aging and age-related pathologies. Such an issue appears particularly relevant in the broadly post-mitotic central nervous system (CNS), where non-replicative neurons are restricted in DNA-repair choices and are prone to accumulate DNA damage, as they remain unreplaced over a lifetime. Both DNA injuries and consecutive DNA-repair strategies are processes that can evoke extrachromosomal circular DNA species, apparently from either part of the genome. Due to their capacity to amplify gene copies and related transcripts, the individual cellular load of extrachromosomal circular DNAs will contribute to a dynamic pool of additional coding and regulatory chromatin elements. Analogous to tumor tissues, where the mosaicism of circular DNAs plays a well-characterized role in oncogene plasticity and drug resistance, we suggest involvement of the “circulome” also in the CNS. Accordingly, we summarize current knowledge on the molecular biogenesis, homeostasis and gene regulatory impacts of circular extrachromosomal DNA and propose, in light of recent discoveries, a critical role in CNS aging and neurodegeneration. Future studies will elucidate the influence of individual extrachromosomal DNA species according to their sequence complexity and regional distribution or cell-type-specific abundance.

## 1. Introduction

Extrachromosomal circular DNA (eccDNA/ecDNA), which is highly conserved across yeast, plants and mammals [1], was first described by Alix Bassel and Yasuo Hoota in 1964 [2]. Since their discovery, the understanding of the heterogeneous genomic sources of origin, structural composition and biological importance of these circularized DNA elements has grown enormously, owing to revolutions in technical utilities and modes of molecular characterization. Likewise, apart from the Circle-Seq protocol [3,4], whole-genome sequencing (WGS) in conjunction with computational algorithms such as AmpliconArchitect, which combines the calibrated analyses of copy number variants (CNV) and structural variants (SV), now allow one to reconstruct the fine architecture of locus-specific amplicons and their assignment to an intra- or extrachromosomal focal origin [5]. These high-throughput technical advancements thus provide an essential basis to define the individual genetic signatures of a tissue-specific “DNA circulome” [5,6].

Depending on cell and tissue type and genetic background, these DNA molecules show broad heterogeneity in size and can comprise eccDNAs/microDNAs of several hundred bp (initially denoted as small polydisperse circular DNAs (spcDNA)) or larger circularized DNA entities of several mega bp, also known as double-minute chromosomes and later referred to as ecDNA, the latter of which often contain oncogenes or drug-resistance genes [7,8]. Overall, microDNAs of 200–400bp represent the most frequent subtype within the mammalian repertoire of eccDNA and are suggested to account for up to 84% of the entire circulome [9,10]. MicroDNAs are abundant in all mammalian tissue types so far investigated, including embryonic and largely non-dividing adult brain, and account for ~10–50kbp of uncharacterized DNA per cell [9,10]. To overcome the inconsistencies in the use of the nomenclature assigned to extrachromosomal circular DNA molecules, we will refer, in the frame of this review, to eccDNA as those entities of smaller size (microDNAs) and to ecDNA as those of higher size prevailing in tumors.

According to the initial discovery in tumors, ecDNA is well-established to emerge in hyperploid neoplastic cells [11] but is also found in human genetic instability syndromes such as Fanconi anemia [12,13]. In coincidence, the cell-threatening scenario of “chromothripsis” (chromosome shattering), a crisis-like event of genomic instability observed particularly in tumor cells and in the germline, can give rise to ecDNA [14,15,16]. Such incidence usually entails the structural decomposition of a chromosomal arm or other chromosomal moieties, followed by fundamental intra- or inter-chromosomal rearrangements of the fragments [17,18]. Importantly, eccDNA is also detected under physiological conditions, as recently identified for blood leucocytes and muscle tissue [19]. Furthermore, the presence of eccDNA is assumed to undergo aging-dependent dynamics in terms of cellular abundance, size and structural peculiarities, as evidenced from rat lymphocytes and cultured human lung fibroblasts [20].

Apart from such basic knowledge, tremendous novel achievements have rendered this field a compelling issue of current research. However, still ignored is a profiling of eccDNA in the context of central nervous system (CNS) disorders and age-related neurodegeneration, though penetrance of CNS pathologies is tightly associated with hereditary genomic instability, DNA-repair deficiencies and environment-related accumulation of genotoxic events or a combination of these conditions [21,22,23]. With relevance to the occurrence of sporadic cases of neurodegeneration might be, in this context, an early-life exposure to chemical genotoxins that encounter neurons with restricted DNA-repair mechanisms [24,25,26]. Apart from first characterizations of putative tissue-specific signatures of eccDNAs across murine tissues [9], open questions concerning their molecular modes of origin and partitioning into daughter cells, their accumulation and degradation rates, transcriptional capabilities and functional importance beyond oncogene amplification and plasticity remain unanswered. Such important scientific issues are now addressed by pioneering novel work on this topic.

Therefore, in this review, we summarize the current knowledge on eccDNA/ecDNA biology that has particularly grown in the field of tumor development and progression [8,27,28]. Motivated by these discoveries, we delineate why expansions of the field to the CNS context might improve our understanding of how acquired CNV might influence the process of healthy brain aging and age-related CNS pathologies, with the eccDNA catalog putatively operating as a dynamic vector of SV-related CNV over a lifetime.

## 2. Molecular Origin and Biogenesis of ecc/ecDNA

### 2.1. eccDNA/ecDNA Biology—Lessons from Neoplastic Tissue

Extrachromosomal DNA exhibits inter-tissue and inter-cell-type variations [1,29] and has recently been shown in replicative, moderately dividing and broadly post-mitotic tissues [9]. However, a systematic profiling of eccDNA characteristics and abundance across organs over the lifetime and elucidation of their individual molecular modes of origin are not yet realized. In particular, the extraordinary situation of the mature CNS, where a syncytium of moderately replicative glia populations and post-mitotic neurons creates a heterogeneous environment of cellular turnover, genomic stress resistance and repair capabilities, has as yet only marginally been explored.

**Figure 1:**
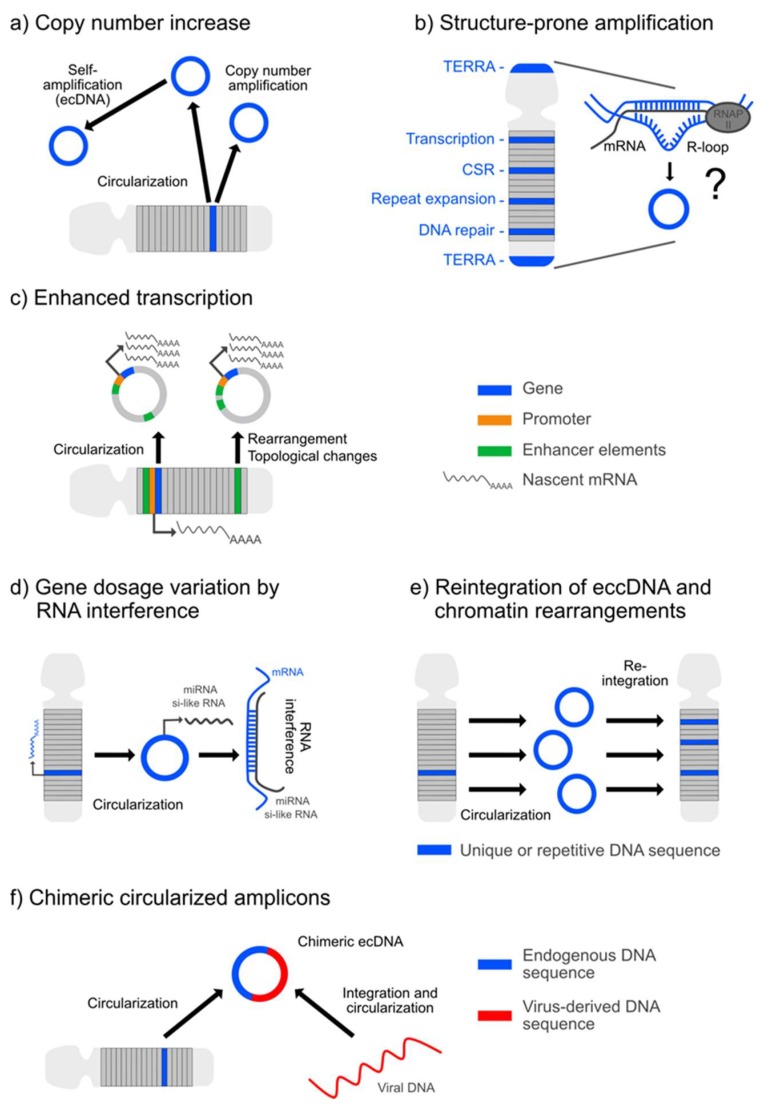
Mechanisms and concepts of extrachromosomal circular DNA (ecc/ecDNA)-derived genomic plasticity. (**a**) Biogenesis of ec/eccDNAs from linear chromosomes leads to a gain in the gene copy number that might be potentiated by self-amplification of the amplicon. (**b**) Several lines of evidence suggest the involvement of R-loops in the generation of ecc/ecDNA. Associated are factors such as: enrichment of transcriptionally active genes on ecc/ecDNAs; transcription-coupled efficiency of class switch recombination (CSR) that generates eccDNA; involvement in repeat transcription, transcriptional termination and DNA instability, as evidenced in several repeat expansion disorders and, thus, in genomic constellations prone to form eccDNA; interaction with different DNA-repair strategies that can give rise to eccDNA; and RNA transcribed from telomere repeats (TERRA) that serve as a marker of ALT, which is characterized by the generation of circular extrachromosomal telomere repeats (ECTRs). (**c**) Enhanced transcription from a gene due to the circularization process, mediated by altered topological influences or by sequence rearrangement on the circle. (**d**) Repressive effects on a gene by the generation of miRNA and si-like RNA sequences derived from eccDNA. (**e**) Genetic rearrangements by the reinsertion of ecc/ecDNA into the linear chromatin. (**f**) Generation of chimeric circularized amplicons consisting of endogenous and viral-derived DNA sequences.

The quantity of extrachromosomal DNA is prominent in cancer [8,27,28], reflecting a situation of complex genomic instability and rearrangements [15]. Owing to an improved molecular profiling, ecDNA is now understood as a crucial oncogenetic plasticity factor [6,7,8] that operates at multiple genomic levels (Figure 1). Apart from the amplification of oncogene copy numbers on ecDNA molecules (Figure 1a), which can influence tumor growth and development, drug resistance and relapse events, the biological role of ecDNA is potentiated by peculiarities in gene expression and transcription rates (Figure 1c), as reflected by the over-representation of ecDNA-encoded transcripts in the tumor transcriptome, thereby reaching levels above values normalized to the absolute gene copy numbers [6,28]. Due to their somatic origin, variations in DNA template and transcript numbers of ecDNA-derived oncogenes are to be scaled to the single cell level, thereby carrying the potential to amplify and accelerate the intra-tumor genomic heterogeneity. Such cell-to-cell variability in the tumor genome might, in turn, facilitate tumor adaption to environmental stressors (e.g., anti-tumor treatment) and propagate tumor progression and drug resistance through the process of selection [6]. Moreover, apart from multiplying oncogene copy and transcript numbers that correlate with the size of the extrachromosomal circles [27], their circularized structure itself is assumed to boost the expression of the ecDNA-hosted oncogene and alter cellular biology, for example, mediated by topology-related regulatory influences [28,30] (Figure 1c). Contextual influence by changes in the genetic environment has recently been demonstrated for the *epidermal growth factor receptor* (*EGFR*) locus, which is co-amplified on ecDNA along with its endogenous enhancers and initial chromatin contacts but receives alternative influence by supplementary noncoding regulatory elements in proximity to promoter and enhancer elements due to the circularized structure of the ecDNA amplicon [31]. Such effects can be multiplied by the implementation or deletion of regulatory elements and their realignment on the amplicon [30] (Figure 1c). Of note, 85.7% of the genome-wide amplifications originate from circularized DNA as assessed by WGS and further confirmed by Circle-Seq, which proves that ecDNA operates as a driver of gene amplification [27]. Thus, the multiplication of oncogenes, such as of *EGFR* and *MYCN* [27,30], on circular chromatin allows them to establish new regulatory catenations and, hence, might bear the potential to drive pro-oncogenic selection [30]. Such ecDNA-based remodeling of oncogenes might follow a dynamic process autonomously sustained by the tumor population, implying a perpetuated mutagenic potential [27]. Accordingly, other genes that are associated with tumor growth but are not *per se* oncogenes, as exemplified for *NTF3* in neuroblastoma, might be enhanced in their expression through copy amplification from ecDNA [27]. In contrast to the aforementioned observations, in a direct comparison, the transcript levels released from genes hosted on small, circularized eccDNA versus linear genes were found to be comparable. Similar results were obtained for an allele-specific expression analysis of circularized copy number-neutral extrachromosomal circles versus their linear counterparts. Thus, DNA circularization alone might not generally be capable of inducing high transcription levels [27] but require regulatory influence from additional factors such as specific enhancer elements, as exemplified for the *EGFR* oncogene [30]. A further yet underestimated influence on the process of rapid tumor diversification and heterogeneity might arise from the discovery that neoplasms, as exemplified for glioblastoma, can amplify identical oncogenes with different temporal kinetics and from different ecDNA molecules, depending on their origin from primary versus relapse tumor tissue [7]. Another important mechanism, which contributes to ecDNA-related tumor heterogeneity, is asymmetric parent-to-daughter transfer of ecDNA that renders cancer cells resistant to internal stress factors and to anticancer approaches [32,33]. Moreover, reintegration of ecDNA into the conventional linear chromosome (Figure 1e), either in an original or rewired sequence, either at sites of tumor-suppressor genes or in proximity to proto-oncogenes or other loci, might influence tumor biology [34]. Of note, the impact of chimeric circularized amplicons integrating elements from different chromosomes or human and viral DNA (Figure 1f), such as of human papillomavirus, as reported in cervical cancer [5], might become a target of future research. In general, ecDNA formation might not only influence the expression of the externalized genes but also impact back on the linear chromosomes by contributing to rearrangements at any position of the intra-chromosomal linear genome (Figure 1e) [27].

Apart, R-loops appear as an interesting structure in the process of ecc/ecDNA biogenesis (Figure 1b), as these triple-stranded DNA:RNA hybrids, involving the misplacement of the single-stranded non-template DNA, are dually involved in DNA damage and DNA-repair processes [35], both of which can promote ecc/ecDNA production. Moreover, as R-loops form during transcription and are determinant in the localization of transcription-associated DNA-damaging events, they might provide a structural link to explain the enrichment of transcriptionally active genes in eccDNA elements. Such considerations coupling R-loops to hotspots of transcription-related gene rearrangements particularly apply to oncogenes [35] but might also have relevance for other genes with high transcription rates. Similarly, R-loops involving telomeric repeat-containing RNA (TERRA) are a signature of the C-circle-generating alternative lengthening of telomeres (ALT) process, which promotes the survival of highly replicative tumor cells through perpetuated telomere restitution. Thus, targeting R-loop formation might represent a genomic approach to restrict oncogene copy number amplification and tumor growth by influencing the amount of ecDNA and extrachromosomal telomere repeats (ECTRs) [35]. However, whether R-loops are the structures that link genic regions to eccDNA excision is still an open issue.

In general, the entire catalog of ecDNA-associated peculiarities in oncogene organization and replication that apparently enable tumors to adapt to selection pressure as imposed by cytostatic treatment or irradiation might, on the other hand, be beneficially repurposed as a strategy for individualized anti-cancer treatment. This comprehensive knowledge provides important, compelling new insights into the genomic regulation of tumor growth by ecDNA (Figure 1).

With its discovery in brain tissue [9], comprising replication-competent glia and post-mitotic neurons, the question arises of a putative analogous role of eccDNA under conditions of genomic instability beyond tumor entities, including genetic background diversities of CNS pathologies.

### 2.2. eccDNA Abundance—A Result of Genomic Instability and DNA Repair

Beyond ecDNA production in tumor tissue, the molecular and genetic processes of eccDNA biogenesis are still not fully understood. As aforementioned, it is suggested that genomic instability, apart from DNA replication and replication-related repair attempts, are primarily involved. Accordingly, augmented eccDNA load is found as a result of double-strand break (DSB) induction [36], which thus might operate as a trigger for eccDNA formation both in a physiological and pathological context.

Of note, several lines of evidence indicate that eccDNA biogenesis does not absolutely depend on homologous recombination (HR) but can be produced by excision from chromosomal sequences, e.g., during the process of HR-independent DNA repair [36]. It has even been suggested that neither HR nor non-homologous end joining (NHEJ; or classical NHEJ, c-NHEJ) are required for eccDNA production and that the eccDNA amount is weakly correlated with the proliferation rate of an individual tissue type [9]. Such discovery sets the basis for an exploration of eccDNA in the broadly post-mitotic tissue context of the CNS (Table 1). Like other cellular entities, neural cell populations harbor individual somatic break points and deletion events, and thus, it appears highly probable that eccDNA contributes to intra-individual genetic mosaicisms also in the CNS [10].

This notion gains support from recent characterizations of the repertoire of DNA-repair mechanisms engaged in eccDNA formation. As a basic proof of principle connecting eccDNA with an HR-restricted neural milieu, Cohen and Lavi demonstrated that eccDNA release from high molecular weight DNA can principally be independent of *de novo* DNA synthesis and HR but will alternatively rely on chromatin excision [36]. This finding is emphasized by the requirement of active DNA ligase IV, a major enzyme necessary to ligate DSB in NHEJ, in the formation of eccDNA in mammalian cells [37]. Accordingly, the broader involvement of HR-independent repair and recombination paths in eccDNA biogenesis has recently been illustrated with the aid of next-generation sequencing technologies coupled to chimeric read mapping strategies in human cell-free plasma [38] and by defining the junction sequences in both ecDNA and eccDNA in bloodstream and tissue [27,38]. According to the junction analyses reported by Zhu and colleagues, eccDNA appears likely to arise from non-replicative NHEJ, as deduced from the frequent joining of blunt ends or very short (1–3bp) microhomology domains, from the process of microhomology-mediated end joining (MM-EJ, also denoted as alternative end joining (a-EJ)), as defined by 4–16bp of homology at the junction ends, as well as non-allelic homologous recombination (NAHR) underlying repetitive elements of high homology (>90%), such as L1 repeats and segmental duplications (SD) or low copy repeats (LCR) [38]. NAHR is assumed to account for ~10–20% of all genomic rearrangements in the human genome [39,40]. Thus, considering that LCR or SD constitute ~5% of the human genome and that their recombination via NAHR poses a threat to genomic stability, this repair mechanism might represent a relevant source for eccDNA generation [41,42]. Accordingly, in plasma, non-replicative repair mechanisms appeared to be strongly involved in eccDNA formation [38], supporting the notion of a similar eccDNA release in the CNS (Table 1).

To generate a comprehensive overview of the DSB-repair cascades engaged in eccDNA biogenesis, chicken B-cell-derived DT40 cell lines with a targeted defect in key DNA-repair molecules operating either in NHEJ, HR or mismatch repair (MMR) were analyzed with regard to their eccDNA abundance [9]. Strikingly, all these mutants retained their capability to produce eccDNA, except for the DT40 MSH3^−/−^ cell line, which displayed a drastic reduction of double-stranded eccDNA by 81%. These data strongly suggest an MMR-mediated mode of eccDNA generation, since the *MutS homolog 3* (*MSH3)*-encoded MSH3 protein is essential in mismatch recognition and repair initiation. In support of these results, the authors conclude that eccDNA generation by mechanisms regularly resulting in threatening mutations might be unlikely but rather arise from repair strategies that reconstitute a normal genome [9]. Overall, short microhomology domains of 4–8bp contributed by 75% to the ligation of microDNA ends [9]. Mechanistically, the authors propose that slippage of the DNA polymerase at microhomology domains will generate single-stranded loops that are excised, circularized and converted into double-stranded eccDNA, with loop excision requiring MMR proteins such as MSH3 [9]. As homozygous mutations in MMR genes cause the mismatch repair cancer syndrome involving brain tumors, this process might have importance for the maintenance of genomic stability also in the CNS (Table 1).

As HR-independent repair paths are of major importance for differentiated neurons to repair DSB, such evidence sets the cornerstone for the generation of eccDNA in largely post-replicative tissues such as the CNS. Neuron-intrinsic restriction of DNA-repair choices arises from the cell cycle phase-dependence of DSB-repair pathways, whereby HR is limited to the availability of sister chromatids in the S and G_2_ phases. NHEJ that requires no or minimal homology (<4bp) can be active in any phase of the cell cycle, including G_0_; however, it is error-prone and increases the risk for persistent gene copy number imbalances and mutagenic events. Of note, even during S/G_2_, NHEJ is assumed to outweigh HR by a ratio of 4:1 [43]. Both the MM-EJ and single-strand annealing (SSA) repair strategies, where short homologous sequences (MM-EJ < 20 bp; SSA > 20 bp) serve for the alignment of broken DNA strands, operate with similar risk of incomplete DSB repair [43,44]. Although expected to be low in G_0_/G_1_ and favored in S/G_2_, MM-EJ and SSA activity might complement NHEJ in DSB repair in post-mitotic neurons, at least in those that can aberrantly reactivate an early S phase [21,45,46,47,48]. This is similar to their up-regulation in HR-negative tumor cells, as these processes are operating not only after but also prior to DNA replication [43,44]. A further, yet unproven, option for CNS neurons might represent the MMR system, as delineated above [9], which purposes the correction of wrongly paired bases either arising from errors in replication and recombination or during the DNA-damage response (DDR) [49,50]. Indeed, adult brain cellular extracts enriched for neuronal nuclei have been shown to express several MMR proteins [51], which are also assumed to play a crucial role in the propagation of repeat expansions in non-replicative cells and, thus, in the generation of several neurodegenerative disorders, including Huntington’s disease (HD) [52]. In summary, though limited in HR, the DSB repair pathways prevailing in the CNS and, particularly, in post-mitotic neurons, all appear likely to contribute to eccDNA biogenesis (Table 1).

Moreover, incomplete repair or non-repaired demise that will accumulate in non-replaceable neurons over time might pose further predilections for the acquisition of somatic break points, even without the presence of a heritable susceptibility factor, and thus, render the CNS a preconditioned environment for tissue-specific eccDNA abundance. Such susceptibility might be potentiated by a continuous exposure of the CNS environment to elevated oxidative stress levels due to the high metabolic activity of neurons, which do not only cause base oxidations but also single-strand breaks (SSB) and DSB [53] (Table 1). Apart from non-dividing neurons, the brain harbors glial populations with reactive mitotic potential, which can additionally contribute to HR-related eccDNA formation and account for events of eccDNA generation by break-independent replication errors. Moreover, it is now well-established that, under certain stress conditions, post-mitotic neurons can reactivate an abortive cell cycle at least up to an S phase that is entailed by partial or complete DNA replication [45,46,47,48]. Whether such events are also a source of eccDNA production, e.g., by the induction of DNA-repair mechanisms reactive to the sensing of DNA-content alterations, is still not explored.

Concrete evidence for eccDNA production in the CNS from excised genomic loci was recently provided by Shibata and colleagues [10]. When characterizing eccDNA during murine brain development, they found eccDNA with a size of 200–400 bp at the highest frequency, with 98% remaining below a size of 1kbp. Generally, the ratio of these microDNA circles to higher size circles was estimated to be 50:1 [10]. Electron microscopy identified both single- and double-stranded eccDNA, apparently with similar prevalence. Single-stranded eccDNA was proposed to originate from ligated Okazaki fragments or from DNA produced in surplus along with replication slippage, or from the digestion of double-stranded nicked DNA circles by nuclease [10]. Overall, microDNA structures were found enriched in the 5′ untranslated region (5′-UTR) of genes, in exons and in CpG islands. In 37% of the eccDNA entities, the eccDNA junctional regions displayed short microhomology repeats of 2–15 bp in length, which were more than 10-fold above a random calculation model [10]. Thus, in general, the finding of excised elements forming eccDNA in the developing brain are in high accordance with recent data characterizing eccDNA in a broad panel of replicative and post-mitotic tissues, including the adult brain, which is composed of occasionally replicative glia and post-mitotic neurons [9].

Moreover, the recent profiling of eccDNA in muscle supports a role of eccDNA in post-mitotic, regeneration-competent tissues. Among a catalogue of ~100,000 different eccDNAs identified in muscle tissue from 16 healthy male donors that apparently originated from all chromosomes, 8.1% of the eccDNA reads mapped to rDNA, 3.5% to long interspersed nuclear elements (LINEs), 3.1% to short interspersed nuclear elements (SINEs), 1.2% to satellites and 0.8% to telomeres [19]. Whether the prevalence of repetitive elements such as telomeric and interspersed sequences arises either from HR or, in light of the low proliferative activity of differentiated non-damaged muscle, from a continuous excision of repetitive sequences from the genome, requires further investigation [19].

Considering such evidence, deletions of any chromosomal origin, including spontaneous deletions or those arising from DNA-repair mechanisms, might give rise to eccDNA both in replicative and post-mitotic tissues and organs.

## 3. Sequence Specificity and eccDNA Abundance

### 3.1. Structural Preponderance

As delineated above, eccDNA biogenesis is related to cancer and genomic instability. Sequence-specific structural predispositions for eccDNA formation represent a further level of intensive research. As an apparently conserved feature, virtually any part of the genome might possess the potential to circularize and form eccDNA [19,54], implying that genic, intergenic and noncoding regions contribute to eccDNA production, including unique and repetitive sites [9,10,55].

Segmented analyses of individual chromosomes, however, revealed a positive correlation between microDNA release and the density of genes and guanine-cytosine (GC)-rich sequences, suggesting that such loci might represent eccDNA hotspots and that genome-wide eccDNA production might pursue a non-random pattern [9]. In support, both ecDNA from tumor cells and eccDNA that derived from normal tissue were found to be substantially enriched in genic regions [9,19,27]. Thereby, larger ecDNAs harbor entire genes to a higher percentage than smaller eccDNAs, which predominantly contain gene fragments [27]. Concrete estimates on the genome coverage of eccDNA reported from recent whole-genome analyses in yeast, however, are commented as putative underestimation due to the preponderance of unique eccDNAs that might have low inter-individual overlap [56]. This is reflected by the reported eccDNA coverage of 23% of the total yeast genome, wherein the entire amount of unique eccDNAs originating from individual out of several yeast populations amounted up to 72% [56]. Moreover, in a comprehensive approach screening eccDNA in a wide panel of tissues and cell lines, shared characteristics included GC content above the genome average and an over-representation of genic regions, particularly of 5′-UTRs of genes, exons and CpG islands [9,10]. Further enriched were segments harboring full-length LINE-1 retrotransposons and those associated with RNA polymerase II, suggesting that active transcription and associated DNA:RNA hybrids, or R-loops, might pose a structural propensity for eccDNA biogenesis [9] (Figure 1b).

As aforementioned, repetitive DNA elements such as telomere repeats and satellite DNA, as well as rDNA and transposon-related repetitive elements, including long terminal repeats (LTRs), are frequent sources of eccDNA [9,19,37,57]. Overall, it was assumed that eccDNAs map to repetitive elements in up to 40–50%, according to the representation of repetitive structures in the entire murine genome [9]. Within the different categories of repetitive elements, however, some subclasses might be over-represented. Likewise, in a recent study setting the focus on retrotransposons as a class of repetitive elements, the Ty1 subfamily of LTR retrotransposons was found over-represented among yeast eccDNA [58]. When assessed in mice, SINEs and LTRs were suggested to contribute equally to the eccDNA load irrespective of the originating tissue (e.g., brain, heart and skeletal muscle, kidney, spleen, lung and liver), whereas LINEs appeared to be enriched particularly in sperm [9].

In spite of repetitive elements apparently representing susceptibility loci for DNA break points and looping-out events, it remained not fully clarified whether repeat elements were over-represented relative to the global content of repeats within the individual genome. To conclusively resolve this question, a genome-wide approach served to compare eccDNA abundances and read identities between the evolutionarily compacted genome of pigeons (~1.11 Gb) in relation to the less-condensed and larger human genome (~3.23 Gb), which contains ~10-times higher amounts of repetitive elements as compared to the pigeon genome [54]. Considering such multipliers, the authors found that repetitive elements as a putative source of eccDNAs are not *per se* over-represented but manifest in proportion to the overall abundance of repetitive elements within the genome, which is dependent on the overall genome size [54]. Sub-analyses revealed that, within the eccDNA reads that mapped to repetitive sequences, the classes of LINEs and LTRs were slightly concentrated in pigeons, whereas, in humans, the classes of simple repeats, SINEs and satellites were enriched relative to expected values. The assumption of a linear representation of eccDNA containing repetitive elements in proportion to the entire genome size is consistent with other studies [9].

Several aspects, particularly the densification of exons and gene-dense regions in eccDNA but also the notion that R-loops might imply a preponderance for eccDNA formation and the aforementioned association with MMR strategies, strongly suggest a role of transcription in the process of eccDNA biogenesis (Figure 1b). In a recent study addressing the relation between eccDNA production and transcription, eccDNA mapping to promoter regions was found to be more than 10-fold enriched over random values. This observation coincided with an enrichment of intron-exon junctions and chromatin marks of active transcription. The authors concluded that RNA transcription and consecutive pre-mRNA splicing are amplifiers of eccDNA production [9]. Of note, R-loops are proposed to be a risk factor for DNA damage due to the single-stranded nature of the displaced DNA strands or due to interference with the DNA-replication process, followed by the induction of recombination-directed repair pathways [59,60].

### 3.2. eccDNA Structures Related to Function

In humans, eccDNA has been found to originate from some functional hotspots that structurally can comprise either entire genes or solely gene fragments. One such hotspot originates from the recombination of genes coding for the variable regions of the T-cell receptors and of immunoglobulin light chains [61,62]. Additionally, eccDNA production arises from an isotype switch of activated B cell-derived immunoglobulin heavy-chain classes, also called class switch recombination (CSR), involving the induction of DSB and deletions within the loci encoding the constant regions of the heavy-chain locus, which are unrelated to antigen specificity [63]. The strand ends are then ligated via NHEJ. Programmed DSB induction in the switch regions involves active transcription and replication origins that are determined by R-loop formation [64] (Figure 1b). To what extent R-loops, which are essential for efficient CSR [65], contribute to eccDNA genesis in the process of CSR has to be explored. Thus, eccDNA is a by-product of genetic rearrangements essential for the establishment of the defense function of the adaptive immune response.

At this point, the question arises as to the extent that eccDNA can modulate self-tolerogenic immune functions and autoimmunity, which are crucial in the immune evasion of tumor cells and in the development of autoimmune spectrum disorders. Of note, CSR of antibodies directed against myelin epitopes were shown to play a critical role in the induction and progression of nervous system deficits in an autoimmune-mediated human myelin oligodendrocyte glycoprotein (hMOG)-based experimental model of multiple sclerosis [66]. Moreover, though not derived from immune effectors but from pathogens themselves, it has recently been demonstrated in insects that circular viral DNA templated from the defective virus genome post-infection is a source of siRNAs that modulate antiviral immunity towards a protective function [67] (Figure 1d). It will be interesting to explore whether the profiling of eccDNA amplicons generated by a recombination in genes guiding adaptive immunity, either acquired via B- or T-cell adaptions, might help identify antigens involved in autoimmune spectrum disorders such as multiple sclerosis.

### 3.3. Telomere-Specific eccDNAs

Telomeres, the end caps of chromosomes, are nucleoprotein complexes comprising multiple copies of tandem TTAGGG repeats and a hexameric protein complex, the shelterin or telosome complex. Molecularly, they terminate in a single-stranded G-rich overhang of 50–400 nucleotides at the 3′ terminal (G-strand), whereas the complementary 5′ end is C-rich (C-strand). Telomere repeat binding factor 2 (TRF2), a component of the shelterin complex, is auxiliary in the telomeric DNA to form a secondary structure, called telomere loop or T-loop, by invasion of the single-stranded 3′ overhang of the G-strand into the double-stranded telomeric DNA [68]. TRF2 together with the DNA-binding protection of telomere 1 (POT1), a further shelterin component that specifically interacts with the single-stranded overhang, prevent the ataxia telangiectasia-mutated (ATM) signaling and ataxia telangiectasia and Rad3-related (ATR) pathways to elicit a DDR at the chromosomal ends [69]. The special interrelations of the DDR and telomere structures have been excellently reviewed [70].

In dividing cells, erosion of telomeres occurs gradually with each round of cell division, thereby inducing senescence. Loss of telomeric DNA can be counteracted by the telomere replenishing enzyme telomerase, implementing a human telomerase reverse transcriptase enzyme (hTERT) and a telomerase RNA (hTR) component, the second of which serves as a template for telomere sequence synthesis [71,72].

In telomerase-negative tumor cells, however, telomere attrition can be counterbalanced by a telomerase-independent telomere restitution process, referred to as the alternative lengthening of telomeres (ALT) [73], the principal mechanism of which is assumed to rely on a DNA-copying step primarily dependent on HR, though it is likely to involve additional molecular mechanisms. Accordingly, any telomere-length maintenance strategy that operates independently of the telomerase enzyme was proposed to be categorized as ALT [74]. Although ALT is reported mainly as a telomere-maintenance mechanism specifically in tumor cells lacking telomerase enzyme activity, it is also active in murine somatic cells [75]. One phenotypic identifier of ALT-positive cells represents the occurrence of telomere-specific eccDNA, termed extrachromosomal telomeric repeats (ECTRs), comprising, apart from linear double-stranded DNA, double-stranded T-circles and single-stranded 5′ C-rich C-circles. Further distinct features include hypervariability of the telomere length, the presence of ALT-associated promyelocytic leukemia bodies (APBs) harboring telomeric chromatin and elevated levels of long noncoding TERRAs that also control telomere lengths [76,77] (Figure 1b). These TERRA transcripts localize to critically short telomeres, where they form DNA:RNA hybrids, or R-loops, that activate the DDR and promote telomere restitution via telomeric sister chromatid exchange (T-SCE) [78]. Thus, increased T-SCE is another signature of ALT. An interesting and still open question, in analogy to the aforementioned, is whether TERRA-formed R-loops are a susceptibility region for ECTR secession and how they influence ALT.

Accumulation of ECTRs occurs, as currently assumed, in 10–15% of the cancers that maintain the telomere length by a telomerase-independent ALT pathway [79]. Molecularly, ECTR in the form of double-stranded T-circles is generated by T-SCE via HR, thereby originating from the resolution of T-loops. These circular ECTRs then provide a template for the expansion of telomere tracts by a rolling circle amplification mechanism [79]. Moreover, T-circles can also originate as a result of HR-mediated telomere rapid deletions (TRD) evoked by dysfunctional TRF2. Apart from repressing DDR signaling at the telomere, TRF2 is essential for the formation of telomeric T-loops, and it prevents their excision by blocking the DNA-repair proteins Nijmegen breakage syndrome 1 (NBS1) and X-ray repair cross-complementing 3 (XRCC3) [80]. A further factor essential for telomere replication, which operates in association with the MRE11-RAD50-NBS1 (MRN) complex, is the C-terminal binding protein (CtBP)-interacting protein (CtIP). Absence of this DNA-repair protein leads to a drastic reduction of telomere length, increased generation of T-circles, DNA damage and chromosomal aberrations [81]. A similar role in telomere replication, maintenance and repair applies to SAE2, the yeast homolog of CtIP [82,83,84]. Interestingly, the inducible loss of CtIP, as investigated in hTERT-immortalized epithelial RPE1 cells derived from the retina, leads to the accumulation of T-circles but not of C-circles [81], the second of which has recently been established as a specific marker of ALT [85]. However, silencing of CtIP in the ALT-positive U2OS cell line resulted in a two-fold increment in C-circles, inferring that CtIP protects telomeres in both scenarios, i.e., a telomerase-positive as well as an ALT-positive environment, and suppressed the release of ECTRs [81].

Previously, Hande and colleagues reported telomere shortening and an accumulation of ECTR DNA in fibroblasts of ataxia-telangiectasia patients and *Atm^−/−^* mice [86]. Along with nuclear ECTR DNA signals, cytoplasmic signs of telomeric DNA were detected, though the exact structure of these ectopic ECTR entities was not defined [86]. A nuclear-to-cytoplasmic translocation of linear and circular ECTRs has also been described in ALT-positive cancer cells [79]. Although the role of ATM and its yeast homolog TEL1 in telomere maintenance is well-established [87], it is not yet clear if it directly contributes to the generation and accumulation of ECTRs, e.g., by TRD processes. Such studies, however, provide a link between extra-nuclear eccDNA and the DNA-sensing cyclic GMP-AMP synthase (cGAS), together with the class of stimulator of interferon genes (STING) (cGAS-STING) pathway, which elicits an interferon-mediated inflammatory reaction in response to highly immunogenic misplaced DNA [88,89]. The functional interconnection between ECTR and cGAS-STING might have potential in the surveillance of ALT-related tumor development, as the ectopic accumulation of ECTRs in telomerase-positive immortalized human fibroblasts expressing a mutant *TRF2* allele reduced cell proliferation by the activation of interferon-responsive cytosolic DNA-sensing pathways [79]. Whether an analogous mechanism of ectopic ECTR accumulation in combination with cytosolic DNA-sensing signaling cascades will also operate in normal human somatic cells deserves further investigation.

Telomere lengths in human cancer cell lines, in human embryonic stem cells (hESCs) and human induced pluripotent stem cells (hiPSCs), all of which overexpress hTR, are further controlled by a process that trims over-elongated telomeres [76,90]. Thus, telomere-length homeostasis is apparently maintained by a balance between telomerase-dependent telomere elongation and a telomeric cropping mechanism that will operate independently from replication-associated telomere attrition. The telomere-trimming process activated as a response to over-extended telomeres in a telomerase-positive cellular milieu results in circular ECTR formation and shares features with the ALT process, such as telomere-length heterogeneity, though APBs remain an inconstant feature [76,90]. Moreover, these cells apparently do not exert T-SCE, which is, by contrast, upregulated under ALT. Although telomere trimming in hESCs and hiPSCs involves the MRN complex member NBS1 and XRCC3, which either participate in or prepare HR, the telomere-cropping mechanism in these cells is described to occur independently of the activation of HR [76]. Thereby, XRCC3 and NBS1 mediate telomere curtailment via the formation of single-stranded C-rich telomeric DNA and double-stranded T-circles derived from T-loops, respectively. Apart from hESCs and hiPSCs, ECTRs were also suggested to originate from telomere-trimming processes in normal neural cells and neuroblastoma cell lines [91].

As aforesaid, another important type of circular ECTR is represented by C-circles. Though specific technical tools and detection assays are now available to investigate ALT activity using C-circles as a specific marker for ALT activity [85], the molecular basis for their synthesis in ALT-positive cells is poorly understood. More recently, a putative mechanism underlying the biogenesis of C-circles was suggested in ALT-positive U2OS cells [92]. Hu and colleagues showed that the partial inhibition of telomerase activity in telomerase-positive human fibrosarcoma HTC75 cells exhibiting telomeric DNA damage leads to the generation of C-circles and APBs, which points to the possible switch from telomerase-positive cancer cells to cells exerting ALT-like mechanisms [93]. Mechanistically, as a result of endogenous telomere DNA damage, SSB and DSB induce replication fork collapse, which triggers the NHEJ-dependent release of C-circles from the lagging strand and C-rich overhangs from the leading strand. These observations imply that the production of C-circles and C-rich overhangs are linked to DDR and replication stress at the telomeres. The HR-independent release of C-circles via NHEJ might also suggest the involvement of a telomere-trimming process for the development of C-circles, whereas telomere elongation via T-SCE characterizes active HR at telomeres [92].

Moreover, C-circles and C-strands were recently detected in the spinal cord and cerebellum, suggesting that telomere-trimming mechanisms [91], or ALT-like processes, may exist in the CNS, which comprises post-mitotic neurons and occasionally replicative glia cells. Whether these mechanisms and ALT-like processes, which need HR-independent operations, will occur in neurons requires future investigation. In general, the load of ECTR in individual tissues might depend, at least partially, on its replicative index, since replication stress propagates the generation of telomere-related C-circles, as shown for hESCs harboring extended telomeres due to the over-expressing hTR. However, the compromised telomere stability of extra-long telomeres might equally add to this observation under the conditions applied [76].

As aforementioned, apart from externalization, eccDNA/ecDNA can also cause genomic rearrangements by insertional events. Analyses of reintegration sites revealed that circle reinsertions can be placed proximal to the *telomerase reverse transcriptase* (*TERT*) gene and, thus, might enhance *TERT* expression [27].

## 4. Replication, Segregation and Degradation of eccDNA

### 4.1. Self-Replication and Self-Limitation of eccDNA Production

The tissue-specific load in eccDNA might not only be a passive consequence of DNA replication, DNA integrity and repair, as delineated before, but also implicate a self-determined regulation. Accordingly, about 80% of all eccDNAs in yeast were found to possess either an origin of replication, an autonomously replicating sequence or at least its related core consensus sequence, as shown by several studies and approved for histone genes in *Saccharomyces cerevisiae* [56,94,95]. Likewise, in the case of tumors, ecDNAs autonomously self-replicate and, thus, contribute to oncogene amplifications [96]. Such self-replication might account for a stable level of eccDNA in a given tissue and organ and influence, if present, its regulatory role on gene copy numbers, e.g., in tumors, neurodegeneration and aging. Likewise, extrachromosomal rDNA circles (ERCs) that encode for ribosomal RNA accumulate in mother cells by asymmetric segregation during mitosis and are associated with mechanisms that propagate yeast aging and reduce lifespan [97]. Apart from self-amplification capacity, optimal eccDNA amounts and related gene copy numbers will therefore also require a restriction control mechanism.

Addressing intrinsic control mechanisms of eccDNA abundance, it has been demonstrated in *Saccharomyces cerevisiae* that the copy number of ERCs is under a tight self-limiting regulation, implicating the potential to activate a need-adapted clonal expansion of ERCs, which will be reintegrated in an amount required to keep cellular homeostasis. Accordingly, a direct correlation between the production and deletion of ERCs was illustrated. Thus, ERCs are considered as a dynamic pool exploited for the rapid adjustment of gene copy numbers to physiological needs [98]. Maintenance of an optimal ERC steady state appeared unlikely to be an inherited feature established under selection pressure, as ERCs are, in most cases, not transferred to daughter cells but almost entirely retained in the mother cell during cell division. Such data strongly support the notion that eccDNA might not only indicate genomic instability or an aberrant chromosomal state but also imply beneficial roles relevant in health maintenance and pathology prevention. Thus, these findings appear of importance in light of the presence of eccDNA also under healthy conditions. Further studies are necessary to explore the existence of a relay mechanism that allows for steady-state levels of eccDNA in a tissue-specific manner also in higher vertebrates.

### 4.2. Cellular Segregation

Due to their acentric structure, eccDNAs segregate unequally during cell division and, thus, can contribute to somatic mosaicisms of copy number variations [33]. Currently, the mechanisms that control eccDNA partitioning during cell division are not fully clarified. In a recent study in yeast, Denoth-Lippuner and colleagues proposed a model of asymmetric circular DNA segregation involving the operation of the multifunctional Spt-Ada-Gcn5 acetyltransferase (SAGA) complex that has prominent roles in posttranslational histone modification, transcriptional elongation and mRNA export [99]. In yeast, SAGA appears required to confine non-chromosomal circular DNAs to the mother cell and prevent segregation into daughter cells during mitosis by mediating their tethering to nuclear pore complexes (NPCs), the transport channels within the nuclear envelope. The authors show that this process of eccDNA attachment to the NPC, exemplified for ERC, involves the transcription and export complex (TREX)-2 of the pore, as well as the multicomponent SAGA complex in its role as a recruitment factor of chromatin. As a result of SAGA-mediated chromatin targeting, the circular DNA in conjunction with the ERC-loaded NPC will be retained in the mother cell. This asymmetric segregation both of ERCs and NPCs will promote cellular aging and shorten the lifespan, with ERCs being the drivers of NPC accumulation. By contrast, ERCs alone were insufficient to accumulate or to promote aging and modulate lifespan. Of note, the authors stated that ERC require anchoring to a moiety of nucleoporins (Nups) that are stably integrated at the NPC, such as those of the core region [97]. It will be interesting to explore whether eccDNA tethering to NPC structures is also a mechanism to modulate eccDNA load in mammalian cells (Table 1) and to define the impact of its deregulation on the development of diseases, which implicate disturbed NPC-associated functions, such as evidenced for *C9ORF72*-related amyotrophic lateral sclerosis (ALS) [100,101,102].

### 4.3. eccDNA Degradation

The turnover of eccDNA within cells or in cell-free DNA of the circulation has not yet been investigated. Cellular DNA is partly degraded by TREX1 (DNA 3′ repair exonuclease I) [103,104]. Generally, mutations in at least five gene loci are identified in humans to be associated with defective nucleic acid metabolism, wherein particularly that coding for TREX1 is evidenced to be important for the clearance of cytosolic DNA species derived from endogenous retro-elements [105,106]. Elimination of extrachromosomal genetic elements has also been associated with structures termed micronuclei. In particular, it was shown that micronuclei were enriched in ecDNA after low-dose exposure to the DNA replication inhibitor hydroxyurea, and this was also associated with a loss of amplified genes on ecDNAs [107]. Furthermore, the entrapment of ecDNAs with amplified *N-myc* was found in micronuclei from neuroblastoma tumor cells *in vivo* [108]. Whether this mechanism applies to other cell types remains to be elucidated.

Generally, cell-free DNAs display short half-lives of several minutes to hours and are eliminated from the blood system by the liver and kidneys [109]. The DNA of a circularized structure shows resistance towards digestion by exonucleases and RNases and, therefore, is more persistent than linear DNA or RNA after release into the blood [110]. Interestingly, most of the extracellular DNA is enclosed in large extracellular vesicles, which contributes to both their dissemination and stability [111]. Hence, results from ongoing and future studies will further shed light on the intra- and extracellular dynamics and turnover rates of eccDNA and contribute to define their utility as “liquid biopsies”, as discussed below [112].

## 5. eccDNA in CNS Aging and Neurodegeneration

### 5.1. Role in CNS Aging

EccDNAs have long been associated with aging in yeast and mammalian cells and tissues [20,29,95]. In particular, more than 20 years ago, Sinclair and Guarente provided evidence that ERCs accumulate in old yeast and might be causal for fungal aging [95]. Moreover, eccDNA abundance has even been proposed to serve as a putative index of cellular aging, as they were found to be amplified during aging in senescence-resistant SAM-R mice, a process that was accelerated in the senescence-prone SAM-P progeria model [113]. In support of this finding, mutations in the *sgs1* gene, the yeast homolog of the Werner’s syndrome gene, lead to a stronger ERC load, associated with characteristics of premature aging and reduced lifespan [95]. In addition, the study by Sinclair and Guarente proposed that the replication of eccDNA in the S phase of the cell cycle, where it was assumed to increase in an exponential manner, might represent the clock that determines the lifespan in yeast. The rise in ERC numbers also correlated with the exponential rise in the mortality rates in yeast [95].

Likewise, in a recent study, the effects of temporal ERC accumulation on the replicative lifespan (RLS) were determined in yeast by using a microfluidics-dependent real-time approach [114]. At the single cell level, the authors longitudinally assessed the yeast cellular vitality and RLS as a function of ERC accumulation. Accordingly, they divided the entire lifespan of each single cell into three major phases related to the senescence entry point (SEP), thereby defining pre-SEP, SEP and post-SEP. Intriguingly, exponential ERC production eventuated before the onset of SEP in the life of mother cells. In this pre-SEP phase, ERC accumulation in the nucleolus was accompanied by an increment in rRNA levels, due to the transcription of ERC-related rDNA. However, the production of functional ribosomes from these rRNAs remained defective. Thus, the miscoordination between abundant rRNA and ribosome biogenesis led to nucleolar stress and impaired the nuclear homeostasis and cellular growth in the post-SEP phase, which might ultimately lead to cell death. Moreover, post-SEP mother cells underwent asymmetric segregation of ERC due to an unequal partitioning of the nucleolus and nucleoplasm, which might serve as a mechanism for the rejuvenation of daughter cells [114]. Hence, this study defined a series of events that might explain how ERC accumulation is associated with cellular aging and death.

Another study highlighted the relationship between transcriptional activity and ecDNA formation in the context of aging. In aging budding yeast, the generation of protein-coding eccDNAs was illustrated to be triggered by the transcriptional stimulation of certain genes sensitive to environmental stimuli [115]. Likewise, in yeast cells that aged under copper sulfate treatment, the *CUP1* gene was transcribed in a site-specific manner, exclusively leading to the accumulation of *CUP1* eccDNA. Apart from such transcriptional control, the *CUP1* eccDNA load was amplified in the aging mother cell by asymmetric segregation and the retention of eccDNAs [115]. Mechanistically, transcription of the *CUP1* gene resulted in DSB and, consecutively, eccDNA formation via Sae2, Mre11- and Mus81-dependent DSB-repair mechanisms, suggesting the involvement of recombination events [115]. Further work will reveal whether such a direct link between transcription and eccDNA formation also exists in other eukaryotes (Figure 1b).

The structural characterization of eccDNA under aging conditions, as performed in rat lymphocytes and cultured human lung fibroblasts, showed a higher dispersion and increased size of eccDNA species relative to young conditions, as well as roughly a doubling in number [20]. Data related to the aging CNS are currently not available; however, eccDNAs isolated from the E13 brain [10] confirm an enrichment in CpG islands similar to many other tissues. This finding suggests a putative novel mechanism involved in genomic plasticity in healthy aging and age-related diseases. Since methylation of CpG islands is a strong epigenetic marker of biological aging also in mice [116], it is tempting to speculate that age-associated epigenetic alterations might influence the number and function of microDNAs in physiological and pathological brain aging.

A further yet unaddressed link between eccDNA, aging and senescence might arise from the discovery that eccDNA harbors a substantial amount of transposable elements (TEs), including LTR and non-LTR retrotransposons [9,19]. Cellular senescence is described to progress through different stages, with the later phase being characterized by the transcriptional de-repression of TEs, accompanied by a sterile inflammation elicited through an interferon-dependent immune response against cytoplasmic TE DNA [88,89,117]. As delineated before, this interferon-mediated inflammation against highly misplaced DNA further involves the cGAS-STING pathway, which is able to sense particularly double-stranded but also single-stranded DNA in a sequence-independent manner and, thus, can be directed against DNA fragments of any origin, including foreign and endogenous DNA, the second arising, for example, from genomic instability [118]. This pathway, in turn, will activate a paracrine senescence-associated secretory phenotype (SASP) and pro-inflammatory SASP-stimulatory NF-κB cascades and is thus involved in a molecular loop that propagates senescence [88,89]. Given that eccDNA will be amplified and/or accumulate over time, an age-related increase in eccDNA harboring TEs or other sequences like ECTRs, as aforementioned, might be of yet unconsidered relevance for the aging process of the CNS and, via the gene regulatory function of transposons, for several neurodegenerative disease processes [118] (Table 1). Of note, several gene mutations driving impairments in the metabolism of nucleic acids, including the processing and degradation of cytosolic DNAs and DNA:RNA hybrids derived from TEs, can elicit a corresponding interferon-mediated inflammatory syndrome in humans in the form of Aicardi-Goutières syndrome, an inherited infantile encephalopathy [105,106]. Whether the proposed interplay between the cGAS-STING pathway and misplaced DNA will implicate similar responses to circularized DNA is still an open issue.

Thus, variations in the spatial distribution and cellular abundance of transposons related to dynamics in the eccDNA load might provide a further level to explain the cellular mosaicism of aging and senescence. However, studies that specify the age-related eccDNA content in the CNS, under consideration of region-specific peculiarities, are still to be performed, particularly in mammals. Such open issues appear of particular importance, as correlations between eccDNA abundance and organismal aging might differ between species [54].

### 5.2. Role in Neurodegenerative Disorders

Based on the knowledge delineated above, it is tempting to speculate that eccDNA in the CNS might recapitulate properties established from in vitro model systems or other tissues [9]. However, the functional role of eccDNA in CNS neurodegeneration is still not defined. Support for eccDNA abundance and impact particularly in the context of age-related CNS pathologies arises from different aspects. First, eccDNA biogenesis implies heterogeneous molecular origins, including DNA-repair mechanisms active in the HR-limited CNS (Table 1). Moreover, aging modifies the tissue-specific eccDNA profiles. As several neurodegenerative disorders are either age-related or associated with acquired or inherited DNA instability, they might accumulate several susceptibility criteria for eccDNA production. Although still unproven, one striking predisposition, particularly in repeat expansion disorders such as HD, Friedreich’s ataxia (FA) and *C9ORF72*-related ALS [119,120,121,122], might arise from the propensity to form R-loops, which are strongly associated with repeat expansion instability, repression and abortion of transcription and elongation and dually with genomic instability and DNA repair [59,123]. Notably, in non-replicative cells, R-loop formation can also occur independently from the generation of DSB. In the presence of DNA damage, R-loops might be decisive in DNA-repair choices via their influence on the extent of end resection and, thus, determine the efficiency of both HR and NHEJ [35] (Table 1). Moreover, inherited defects in DNA-repair cascades [23] might coincide with the natural limitation of neurons to respond to DNA injuries [21,22] and age- or mutation-related disturbances in nuclear envelope structures associated with the organization of nuclear chromatin [124,125,126]. This can be potentiated by extrinsic factors such as the continuous exposure to DNA-damaging metabolites like ROS and early-life encounters of environmental compounds such as the cycad genotoxin methylazoxymethanol (MAM), the latter of which has been described as a putative contributor to the slow induction of Western-Pacific ALS and the parkinsonism-dementia complex (ALS-PDC) [25,127]. Likewise, Kisby and colleagues showed in mice that MAM treatments lead to *O^6^*-methylguanine DNA lesions in the brain, resulting in an altered expression of genes and molecular signaling cascades linked to developmental and degenerative neurological phenotypes and tumor formations [24]. All of these heterogeneous factors might increase the propensity for eccDNA biogenesis in the CNS (Table 1).

Likewise, in a mouse model of age-related HD, it was shown that DNA damage by ROS-mediated base oxidation propagates the expansion of CAG repeat tracts through a strand slippage event during a base excision repair process [127]. Based on such evidence and data that supported the operation of an analogous mechanism in neuron-like cell extracts [128], a DNA-repair mechanism was proposed to explain the expansion of disease-related microsatellites in non-dividing neurons [52]. According to this model, oxygen radicals will cause a small gap in the repeat-carrying DNA strand, followed by the looping-out of a chromatin strap that will be converted into a stabilized hairpin structure through recruitment of the heterodimeric MSH2-MSH3 complex of MMR proteins. The slipped structure-prone DNA intermediate will finally be resolved and reintegrated by error-prone DNA repair and, thus, contribute to the elongation of the repeat [52]. Accounting for the representation of satellite structures within the catalogue of eccDNAs, one might assume that MMR-related repeat expansions, apart from the MMR process itself, might serve as an amplifier for the generation of circular DNA, particularly in the CNS with perpetuated DNA-damaging ROS levels. This could be investigated either in *human superoxide dismutase 1* (*hSOD1*)- or *C9ORF72*-related genetic models of ALS, which entail a defect in ROS neutralization or disease-associated hexanucleotide repeats, respectively [129].

Another possible link between CNS aging, neurodegenerative diseases like *C9ORF72*-related ALS and eccDNAs relies on the structural and functional properties of the NPC (Table 1). Deficits in the NPC-mediated nucleus-to-cytoplasm transport of RNA and higher molecular weight proteins are assumed to contribute to neuronal dysfunction under healthy CNS aging [124] and play a crucial role in the manifestation and progression of neurodegenerative *C9ORF72*-related ALS [100,101,102]. Apart from operating as a bidirectional nucleo-cytoplasmic transport channel, the NPC assembled from multiple copies of about 30 Nups physically interact with the nuclear chromatin. In yeast, structural vicinity of the NPC basket protein Mlp1/2, the yeast homolog of the human translocated promoter region (Tpr), to chromatin sites of active transcription suppresses the generation of R-loops and, thereby, promotes genomic stability [130]. This suggests that, on the contrary, alterations in NPC basket proteins might propagate R-loop formations and, putatively, the generation of eccDNA. In accordance, R-loops accumulate if yeast Mlp1/2 is lost [130]. Moreover, and with evidence coming from different species, including mice and humans, NPCs were found to contribute to nuclear chromosome organization within the nuclear space and, according to the so-called gene-gating hypothesis [131], to the regulation of certain genes involved, e.g., in cell cycle and development, including neuro- and myogenesis [132,133,134]. In short, this gene-gating hypothesis postulates that the physical approximation of transcribed genes to the NPC facilitates the formation of mRNA ready for nuclear export [131]. Although still understudied in their identity, the Tpr-interacting basket Nup153 and the linker Nup93 were evidenced to mediate gene gating in the human U2OS cell line [133]. In yeast, recruited genes possess gene recruitment sequences [135] and interact with the multifunctional SAGA complex [136,137]. Interestingly, as aforementioned, chromatin recruitment in yeast by the multi-subunit SAGA complex [138] will include ERCs through their interaction with the mRNA export and gene-gating factor TREX-2 of the nuclear pore and result in the tethering of ERCs to a putatively not yet fully defined set of Nups of the NPC [97]. Following cell division, the resulting asymmetric partitioning of NPCs and ERCs, both of which will be retained in the yeast mother cell after cell division, will promote aging and reduce the lifetime [97].

Therefore, exploring a corresponding role of SAGA-dependent anchoring of eccDNA to NPC structures in mammals might open a novel important approach to a better understanding of CNS aging and age-related nervous system disorders, in particular those known to involve NPC dysfunctions such as *C9ORF72*-related ALS. Thereby, in case the notion will hold true, discovering the individual eccDNA anchoring Nups and a further exploration of the functional consequences of eccDNA tethering, in particular in light of gene-gating effects and proper NPC export capacities, are further challenging aspects that merit investigation. Additionally, interesting to resolve is the question of whether there is a sequence-specific selection of eccDNAs, which might have a peculiar affinity to SAGA-mediated NPC attachment. As basket Tpr, the human homolog of yeast Mlp1/2, was shown to regulate the absolute number of NPCs within the nuclear envelope [139], it might constitute one of the putative SAGA targets in mammals. In support of this, in humans, the TREX-2 complex, which interacts with SAGA, has recently been shown to stably localize to the pore basket through interactions with basket Nup153 and Tpr [140]. Moreover, Tpr was demonstrated to be required for proper chromatin distribution in the nuclear periphery by the establishment of nuclear sub-compartments that maintain the NPC-associated heterochromatin exclusion areas in HeLa cells [141].

Another functional interaction might implicate the establishment of retention gradients of eccDNA entities according to the rate of eccDNA replication, transcription or copy numbers beneficial to keeping cellular homeostasis, which will depend on the density of NPC integrated in the nuclear envelope. Of note, it is currently not well understood how far the number and constitution of NPCs are preserved or regulated in post-mitotic neurons over a lifetime, as they will not be replaced as entire transport units but be successively exchanged for their individual sub-complexes [142,143].

Consistent with a notion of SAGA in human neurodegeneration, polyglutamine expansions in the ataxin-7 protein, representing an integral subunit of human SAGA-homologous complexes [144], were considered to contribute to the manifestation of degenerative retinopathy in autosomal-dominant spinocerebellar ataxia type-7 [99,145]. Analogous neurodegenerative features were also found in flies that expressed reduced levels of the ataxin-7 homolog, entailed by a decomposition of the SAGA complex [146]. However, it is unclear how these neurodegenerative features relate to specific functions of the SAGA complex. Thus, exploring a putative role of the SAGA/eccDNA–TREX-2/NPC interaction in mammalians, in analogy to yeast, will further broaden the profile of this multifunctional, highly conserved complex that is now known to participate in histone modification, transcription elongation, protein homeostasis, telomere maintenance and DDR activation [138,147], all functions relevant in aging and neurodegeneration. Interestingly, CSR mentioned before to be a source of eccDNA production at genetic loci coding for immunoglobulin heavy-chain classes was evidenced to essentially rely on SAGA activity for proper DDR signaling and DSB repair following recombination [147].

Since the putative role of eccDNA in CNS neurodegeneration is still completely unexplored, circularization hotspots are not defined. However, there are neuromuscular pathologies, also with CNS infliction, for which diagnostic markers might show a pathophysiological link to circularization hotspots of eccDNA. Likewise, the *Agrin* gene was recurrently detected on genic eccDNA in muscle tissue analyzed from pigeons [54]. The human homolog of this gene encodes for a heparan sulfate proteoglycan that is released by motor neurons to operate at the neuromuscular junction. It binds to the low-density lipoprotein-related receptor protein 4 (LRP4) at the postsynaptic membrane, where it is required for the clustering of acetylcholine receptors and other proteins. Notably, Agrin antibodies are found in autoimmune-mediated myasthenia gravis (MG) and in neurodegenerative ALS, both of which affect the neuromuscular junction [148,149]. Similarly, Titin, whose antibodies serve as a sensitive marker in paraneoplastic thymoma-associated MG, has been identified as a hotspot among eccDNAs in healthy humans and in *Caenorhabditis elegans* [19,55] (Table 1). Further studies are required to explore the clinical relevance of such genic eccDNAs in neuromuscular and neurodegenerative diseases, including autoimmune entities.

Prominent in eccDNA hotspots in terms of repetitive elements is the presence of retrotransposable elements discussed to participate in the pathogenesis of several neurodegenerative disorders, including ALS and autoinflammatory diseases, as well as in the process of aging [150] (Table 1). The coincidence of TE de-repression in the diseased and aging CNS with their abundance in the moiety of eccDNAs harbored throughout the genome argues for a putative disposition of the CNS environment for eccDNA-related gene regulatory influences, as discussed for the aging process.

Still unexplored in the context of repetitive structures is the question of whether the instability of telomeric TTAGGG tandem repeats at deeper telomere sites, e.g., induced by ROS-dependent DNA damage [151,152], might increase the load of ECTR T- or C-circles, e.g., under the deficiency to neutralize ROS.

In a recent study of our own, we analyzed telomere lengths in a mouse model of ALS, underlying the overexpression of a mutant hSOD1^G93A^ enzyme. In these animals displaying increased oxidative stress levels, unanticipated disease stage-dependent telomere elongations were detected in disease-afflicted cerebral cortex and spinal cord regions. Such telomere lengthening was also evidenced when neurons, which do not possess substantial telomerase activity, were specifically analyzed. Such results were in contrast to the telomere-shortening process observed before in aged cortical brain tissue in mice [153]. Given that ALT can occur independently of HR, however, will underlie replication deficits and the generation of replication intermediates, as recently demonstrated in stem cells [76], such telomere-maintenance mechanisms might pose an option to explain the relative increase in telomere lengths in our disease model. This notion is supported by the knowledge that terminally differentiated neurons can re-induce an abortive cell cycle under certain circumstances, including genomic stress and neuropathological conditions, such as ALS, Alzheimer’s dementia and Parkinson’s disease [45,48,154], putatively serving as an unconventional DNA-repair mechanism [154]. This atypical pseudo-mitotic cell cycle will entail S-phase recapitulation and a pathway of DNA replication that was suggested to involve the loading of the repair enzyme DNA polymerase-β onto neuronal replication forks [155], though without the execution of cytokinesis. Unscheduled cell cycle reactivation thus shares similarities with the conditions of replication stress and replication intermediates in proliferative cells. However, it is currently unaddressed whether HR-restricted, DNA-damaged neurons might execute processes similar to ALT in order to maintain telomere lengths over lifetime (Table 1). This question appears of particular importance in light of the fact that ALT processes at telomeres engage several factors that serve as regulatory elements in DNA-repair pathways [156].

Apart from telomeres *per se* representing a fragile site of genome stability due to their secondary structure, including their propensity to form G-quadruplexes, both age-dependent telomere erosion and hyper-elongated telomeres are reflecting states of compromised genomic stability and, thus, might be prone to drive a cell into senescence and apoptosis [157]. Therefore, the region-specific abundance of ECTR might provide valuable information on telomere stability.

## 6. eccDNAs as Diagnostic Tools

Increasing evidence demonstrates the potential application of cell-free DNA (cfDNA) as a candidate marker for the diagnosis, prognosis and monitoring of several diseases, including cancer [158]. The recent finding of extrachromosomal DNA in human blood and plasma under normal and cancerous conditions, and the fact that circularized DNA is more stable than linear DNA, points to a potential suitability as a biomarker [38,110,159]. In support of their value as clinical monitoring tools, and consistent with the fact that microDNAs from lung cancers are larger than that of normal tissue, the circulating eccDNAs in patients prior to surgical tumor resection were found to be enlarged compared to the circulating eccDNAs in the same patients several weeks after surgery [110]. Thus, the use of eccDNA as a “liquid biopsy” might not only imply a diagnostic and prognostic value but also serve to assess treatment efficacy. Additionally, Sin and colleagues identified and characterized eccDNA in pregnant women and observed that eccDNAs were enlarged in comparison to their linear counterparts, whereas fetal eccDNAs were shorter than those of maternal origin [160]. Such findings might contribute to the development of new tools for non-invasive prenatal testing based on stable cfDNA. In analogy to the currently assessed value in tumor diagnostics and surveillance, eccDNA might qualify as a biomarker in the blood and/or cerebrospinal fluid of patients with neurodegenerative disorders. Thus, monitoring the eccDNA repertoire in such accessible compartments under consideration of a sequence-specific profile might gain clinical importance by the putative potential to serve as a predictive or follow-up biomarker in several diseases.

One critical aspect in this context is the differentiation of microDNA from other byproducts, including those arising from apoptotic processes. As shown recently, the identification of eccDNA transcripts and purification of identical eccDNA species from different tissues may help dissect apoptotic fragments from circularized DNA with putative biological importance [19].

An alternative, innovative approach to assess the biological role of eccDNA, other than through the extraction of natural eccDNA templates from cells and tissues, was introduced in a recent study where an exogenous dual-fluorescence biosensor cassette (ECC biosensor) was employed to artificially create eccDNAs [161]. Targeted DNA deletion within the ECC biosensor by means of CRISPR-Cas9 technology generated mono-fluorescent endogenous genic and intergenic eccDNA particles of different sizes following end-ligation of the fragments [161]. Having proven this bioengineering concept to efficiently and specifically induce eccDNA release from different genomic loci, it appears as a highly promising tool to evaluate sequence-specific eccDNA importance in a cell- and tissue-type-specific manner and define effects related to eccDNA stability, abundance and structure. Such a gene-editing strategy related to eccDNA will also elucidate whether eccDNA can serve as a biomarker for genomic stability. This notion appears particularly relevant for the CNS, as neurons are limited in their DNA-repair repertoire.

## 7. Conclusions and Preview

It has been proposed that mechanisms involved in the generation of eccDNA could serve as biomarkers and therapeutic targets in the frame of oncogene-associated pathologies [112]. In analogy, establishing eccDNA as a biomarker in the context of brain aging and age-related CNS disorders might become a further achievement of clinical value, considering the current rise in the average life expectancy and in the prevalence of neurodegenerative diseases. Such expansions, however, will require a detailed molecular and quantitative characterization of eccDNA species, including sub-stratifications according to the disease entity and stage, considering hereditary genetic background parameters and environmental factors that will impact on genomic stability and repair. In support of their usefulness in the field of CNS disorders is the evidence that eccDNA biogenesis might not occur at random but, rather, require certain circularization hotspots, including genic loci. Likewise, there is the recurrence of eccDNA from genes whose products play a role in neurodegenerative and neuromuscular disorders such as ALS and MG. With respect to non-genic sequences, eccDNAs show a well-reproduced association with repetitive elements of different molecular origins. Many neurodegenerative disorders, such as the class of repeat expansions including HD, FA and *C9ORF72*-related ALS, implicate pathomechanisms linked to repeat length and stability. Thus, aligning subclusters of repetitive eccDNA to the scope of inherited disorders manifesting with a neurodegenerative phenotype might be a first step in the transfer of current eccDNA knowledge to the field of CNS pathologies. Such characterizations are likely to give further insight into the pathomechanism of the diseases themselves, as exemplified for the interplay of eccDNA and the NPC in yeast and the pro-inflammatory role of ectopic DNA in humans. As eccDNA is also found in healthy subjects, temporal changes in the eccDNA profile, for example, with respect to CpG islands, which are a strong epigenetic marker of aging, or of telomere-specific ECTR, might serve as longitudinal markers of genomic aging and stability also in the brain.

Realization of such parameters in a clinical context will deserve, as a prerequisite, robust evidence from preclinical studies to allow a first valuable estimate regarding the diagnostic power of eccDNA. Moreover, technical means will be required that allow longitudinal eccDNA analyses by efficient, sensitive and noninvasive approaches, as recently introduced for cfDNA determinations. In support of such perspectives is the well-established use of cell-free linear plasma DNA as highly sensitive parameter for non-invasive prenatal diagnostics. In analogy, monitoring cfDNA, i.e., cell-free circular plasma DNA, might bear a similar diagnostic potential and provide valuable information, for example, on tumor elimination or relapse events and progression rates of neurodegenerative pathologies and, thereby, gain importance in the longitudinal medical attendance of several disorders. Considering the extraordinary stability of circularized DNA due to its resistance against digestion by exonucleases and RNases, such an analysis might outperform linear molecules in terms of sensitivity to serve as a biomarker. In order to assign specificity, further studies will have to explore how far eccDNA arises from DSB that eventuate daily to any cell in the organism and elucidate the means how eccDNA is transmitted from tissue into the circulation, which could occur after the death of a cell or via endosomal transport processes [110].

When defining novel biomarkers, both their predictive (likely treatment benefit) and prognostic (assumed health outcome irrespective of a treatment) strengths are important parameters, as they are well-established in the field of oncology and required for CNS pathologies. Likewise, longitudinal copy number assessments of eccDNA hotspots might entail prognostic information on the aggressiveness of a certain neurodegenerative process, wherein the eccDNA sequence and structural characterization might add to the diagnostic security. Alongside, technical tools such as the AmpliconArchitect [5] that complements WGS data by reconstructing a detailed structure of focal amplicons will gain practical importance. Whether microDNAs will convey predictive values, e.g., in the treatment of autoimmune-mediated disorders such as multiple sclerosis or MG, is yet not explored but, if so, might support decision-making towards therapeutic strategies. Longitudinal assessments and the interpretation of eccDNAs in their function as a biomarker will also require knowledge on their stability in cellular compartments and the circulation and how they are cleared from either location. Such considerations are particularly relevant in light of a yet unproven role of eccDNAs in cellular homeostasis and the possibility that dynamics in eccDNA abundance might beneficially and detrimentally impact on gene dosages. Thus, novel biotechnologies based, e.g., on CRISPR-C-mediated artificial releases of tailored eccDNAs [161] will provide preclinical estimates both on the biological influences as well as the turnover rates of eccDNA in defined tissues and cell types and allow a preview on how attractive eccDNA species might be as candidates for unprecedented molecular biomarkers. The challenging question of whether eccDNA-targeting might also carry therapeutic potential deserves future investigation.

## Figures and Tables

**Table 1:** Processes related to extrachromosomal circular DNA (ecc/ecDNA) biogenesis with putative operation in the central nervous system (CNS) environment.

**Mechanisms Arguing for eccDNA Synthesis in the CNS**		
**Genomic instability**	DSB Oxidative stress Repeat expansions R-loops TEs	
**Main DNA-repair mechanisms related to eccDNA biogenesis** **Telomere-maintenance mechanisms related to ECTR**	Replicative and non-replicative cells	Replicative cells
	NHEJ MM-EJ NAHR MMR ALT-like processes?	HR ALT TERRA
**Genomic hotspots for eccDNA formation (e.g., *Agrin* and *Titin*)**		
**High transcriptional activity in neurons**		
**Chromatin organization via NPC** **NPC dysfunction in CNS aging and neurodegeneration**		

DSB: double-strand breaks, TEs: transposable elements, ECTR: extrachromosomal telomere repeats, NHEJ: non-homologous end joining, MM-EJ: microhomology-mediated end joining, NAHR: non-allelic homologous recombination, MMR: mismatch repair, ALT: alternative lengthening of telomeres, HR: homologous recombination, and NPC: nuclear pore complexes.

## Abbreviations

a-EJ	Alternative end joining
ALS	Amyotrophic lateral sclerosis
ALT	Alternative lengthening of telomeres
APB	ALT-associated promyelocytic leukemia body
ATM	Ataxia telangiectasia mutated
ATR	Ataxia telangiectasia and Rad3-related
cGAS	Cyclic GMP-AMP synthase
cfDNA	Cell-free DNA
CNS	Central nervous system
CNV	Copy number variant
CSR	Class switch recombination
CtIP	Carboxy-terminal binding protein (CtBP) interacting protein
DDR	DNA-damage response
DSB	Double-strand break
ecc/ecDNA	Extrachromosomal circular DNA
ECTR	Extrachromosomal telomere repeat
EGFR	Epidermal growth factor receptor
ERC	Extrachromosomal rDNA circle
FA	Friedreich’s ataxia
GC	Guanine-cytosine
HD	Huntington’s disease
hESC	Human embryonic stem cells
hiPSC	Human induced pluripotent stem cells
hMOG	Human myelin oligodendrocyte glycoprotein
HR	Homologous recombination
hSOD1	Human superoxide dismutase 1
hTERT	Human telomerase reverse transcriptase
hTR	Human telomerase RNA
LINE	Long interspersed nuclear element
LTR	Long terminal repeat
LCR	Low copy repeat
LRP4	Low-density lipoprotein-related receptor protein 4
MAM	Methylazoxymethanol
MG	Myasthenia gravis
MM-EJ	Microhomology-mediated end joining
MMR	Mismatch repair
MRN	MRE11-RAD50-NBS1 complex
MSH3	MutS homolog 3
NAHR	Non-allelic homologous recombination
NBS1	Nijmegen breakage syndrome 1
NHEJ	Non-homologous end joining
NPC	Nuclear pore complex
Nup	Nucleoporin
RLS	Replicative lifespan
POT1	Protection of telomere 1
rDNA	DNA encoding for ribosomal RNA
ROS	Reactive oxygen species
SAGA	Spt-Ada-Gcn5 acetyltransferase
SAM	Senescence associated mice
SASP	Senescence associated secretory phenotype
SD	Segmental duplication
SEP	Senescence entry point
SINE	Short interspersed nuclear element
siRNA	Small interfering RNA
SpcDNA	Small polydisperse circular DNA
SSA	Single-strand annealing
SSB	Single-strand break
STING	Stimulator of interferon genes
SV	Structural variant
TE	Transposable element
TERRA	Telomeric repeat-containing RNA
Tpr	Translocation promoter region
TRD	Telomere rapid deletion
TREX-2	Transcription and export complex-2
TRF2	Telomeric repeat binding factor 2
T-SCE	Telomeric sister chromatid exchange
5′-UTR	5′-untranslated region
WGS	Whole-genome sequencing
XRCC3	X-ray repair cross-complementing 3
